# Supplementary material for: Institutional investors’ site visits and investment-cash flow sensitivity: Mitigating financing constraints or inhibiting agent conflicts?
Source: PLoS One. 2024 Mar 28;19(3):e0300332. doi: 10.1371/journal.pone.0300332 (PMC10977698; doi:10.1371/journal.pone.0300332)
Supplement: S1 Data — (ZIP) [file pone.0300332.s001.zip › Data/result/Table 2 Descriptive statistics.rtf]

Variable	N	Mean	p50	SD	Min	Max	
Invest2 w	9626	0.0580	0.0380	0.0630	-0.0200	0.329	
CF2 w	9626	0.0480	0.0460	0.0850	-0.228	0.324	
vjfreq w	9626	1.138	1.099	0.958	0	3.332	
vjnum w	9626	2.108	2.303	1.704	0	5.371	
Size w	9626	21.95	21.83	1.097	19.86	25.28	
Lev w	9626	0.393	0.379	0.200	0.0500	0.863	
TQc w	9626	2.915	2.276	2.068	0.896	13.10	
RoaB w	9626	0.0410	0.0400	0.0630	-0.250	0.215	
AGE cl w	9626	2.804	2.833	0.334	1.946	3.466	
Finindex	9626	8.267	9.025	1.790	0.450	10.89	
