# Supplementary material for: Institutional investors’ site visits and investment-cash flow sensitivity: Mitigating financing constraints or inhibiting agent conflicts?
Source: PLoS One. 2024 Mar 28;19(3):e0300332. doi: 10.1371/journal.pone.0300332 (PMC10977698; doi:10.1371/journal.pone.0300332)
Supplement: S1 Data — (ZIP) [file pone.0300332.s001.zip › Data/result/Table 3 Pearson correlation coefficients.rtf]

|Inve~2 w	CF2 w	vjfreq w	vjnum w	Size w	Lev w	TQc w		
Invest2 w	1							
CF2 w	0.206***	1						
vjfreq w	0.129***	0.096***	1					
vjnum w	0.151***	0.112***	0.886***	1				
Size w	0	0.0160	0.133***	0.157***	1			
Lev w	-0.027***	-0.196***	-0.059***	-0.072***	0.530***	1		
TQc w	0.045***	0.131***	0.044***	0.107***	-0.492***	-0.358***	1	
RoaB w	0.170***	0.392***	0.245***	0.292***	0	-0.339***	0.221***	
AGE cl w	-0.119***	-0.026***	-0.183***	-0.183***	0.180***	0.167***	-0.102***	
Finindex	0.052***	0.037***	0.127***	0.096***	-0.079***	-0.023**	0.00800	
								
RoaB w	AGE cl w	Finindex						
RoaB w	1							
AGE cl w	-0.071***	1						
Finindex	0.043***	-0.104***	1					
