# Supplementary figures and images for: Institutional investors’ site visits and investment-cash flow sensitivity: Mitigating financing constraints or inhibiting agent conflicts?
Source: PLoS One. 2024 Mar 28;19(3):e0300332. doi: 10.1371/journal.pone.0300332 (PMC10977698; doi:10.1371/journal.pone.0300332)

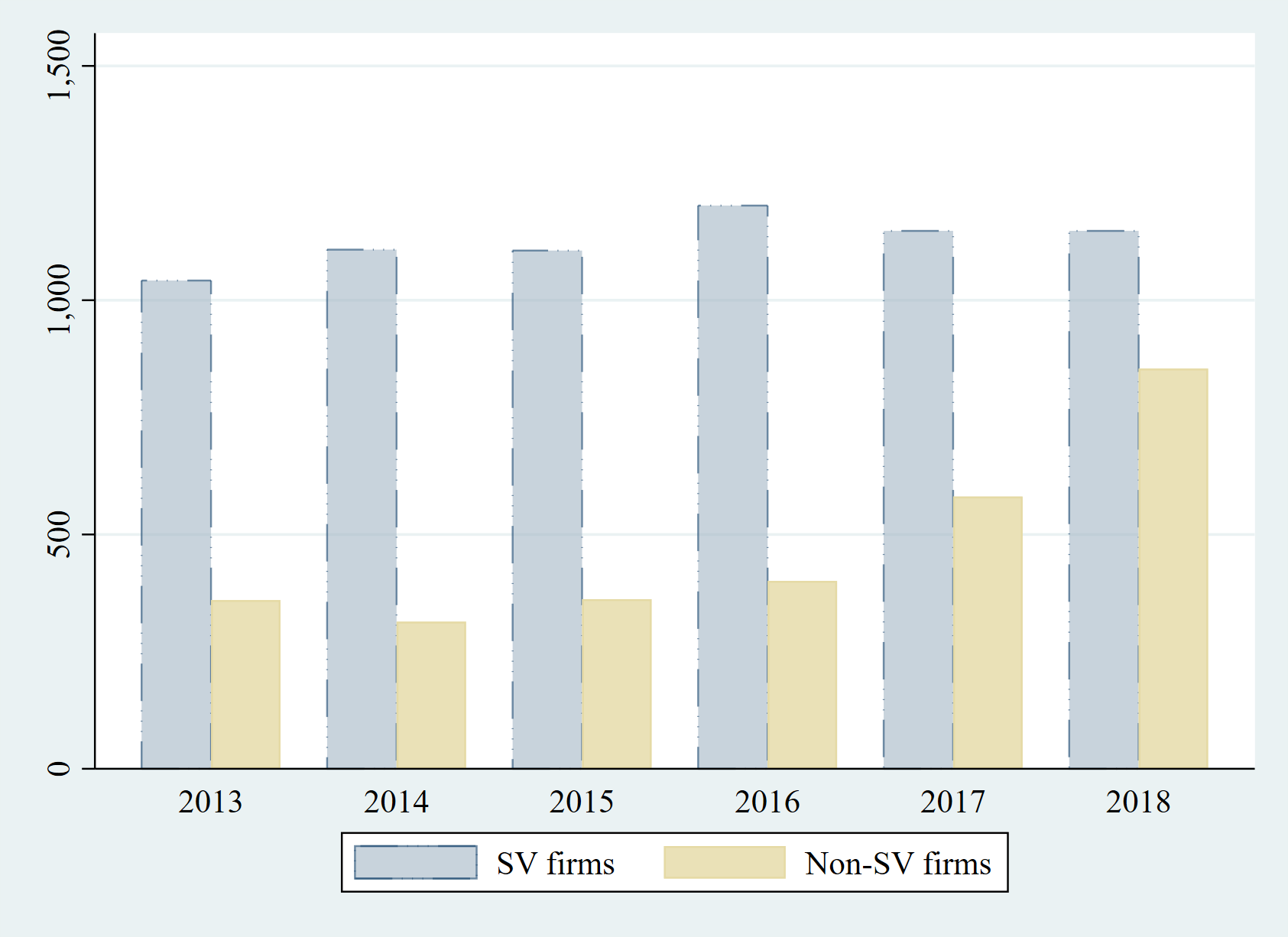

Supplement: S1 Data — (ZIP) [file pone.0300332.s001.zip › Data/result/Figure 1 Sample distribution.png]
