# Supplementary material for: Institutional investors’ site visits and investment-cash flow sensitivity: Mitigating financing constraints or inhibiting agent conflicts?
Source: PLoS One. 2024 Mar 28;19(3):e0300332. doi: 10.1371/journal.pone.0300332 (PMC10977698; doi:10.1371/journal.pone.0300332)
Supplement: S1 Appendix — (DOCX) [file pone.0300332.s002.docx]

**Appendix A: Variable definitions**

**Table A** Variable definitions

| Variables | Label | Calculation methods |
| --- | --- | --- |
| Dependent variable | *Invest* | the ratio of cash payments for fixed assets, intangible assets, and other long-term assets minus cash receipts from selling these assets to beginning total assets |
| Independent  variables | *CF* | the ratio of net cash flow from operating activities to beginning total assets |
|  | *inv_fre* | the natural logarithm of one plus the number of SVs to a firm by all institutional investors during a given year |
|  | *inv_bre* | the natural logarithm of one plus the number of institutional investors to conduct SVs to a firm during a given year |
| Control variables | *Size* | the natural logarithm of total assets |
|  | *Lev* | the ratio of total liabilities to total assets |
|  | *Q* | the ratio of market value of equity plus book value of liabilities to total assets. |
|  | *Return* | the ratio of net profit to total assets |
|  | *Age* | the number of established years of the company |
|  | *Finindex* | the financial development index of the region where the company is registered |
