# Supplementary material for: Institutional investors’ site visits and investment-cash flow sensitivity: Mitigating financing constraints or inhibiting agent conflicts?
Source: PLoS One. 2024 Mar 28;19(3):e0300332. doi: 10.1371/journal.pone.0300332 (PMC10977698; doi:10.1371/journal.pone.0300332)
Supplement: S2 Appendix — (DOCX) [file pone.0300332.s003.docx]

**Appendix B: The details of the PSM procedure**

We divide the sample into treatment group (SV firms) and control group (Non-SV firms) and then conduct 1:1 nearest neighbor matching with all control variables in the model (1) as matching variables, and obtain 8,681 valid observations after matching. Figures 2 and 3 show the density before and after pairing, respectively, we can find that there are significant differences in the characteristics of the treatment and control groups before matching, while the density curves of the two almost overlap after matching. The above results show that the PSM procedure is reliable, and treatment group and control group are better comparable after matching.


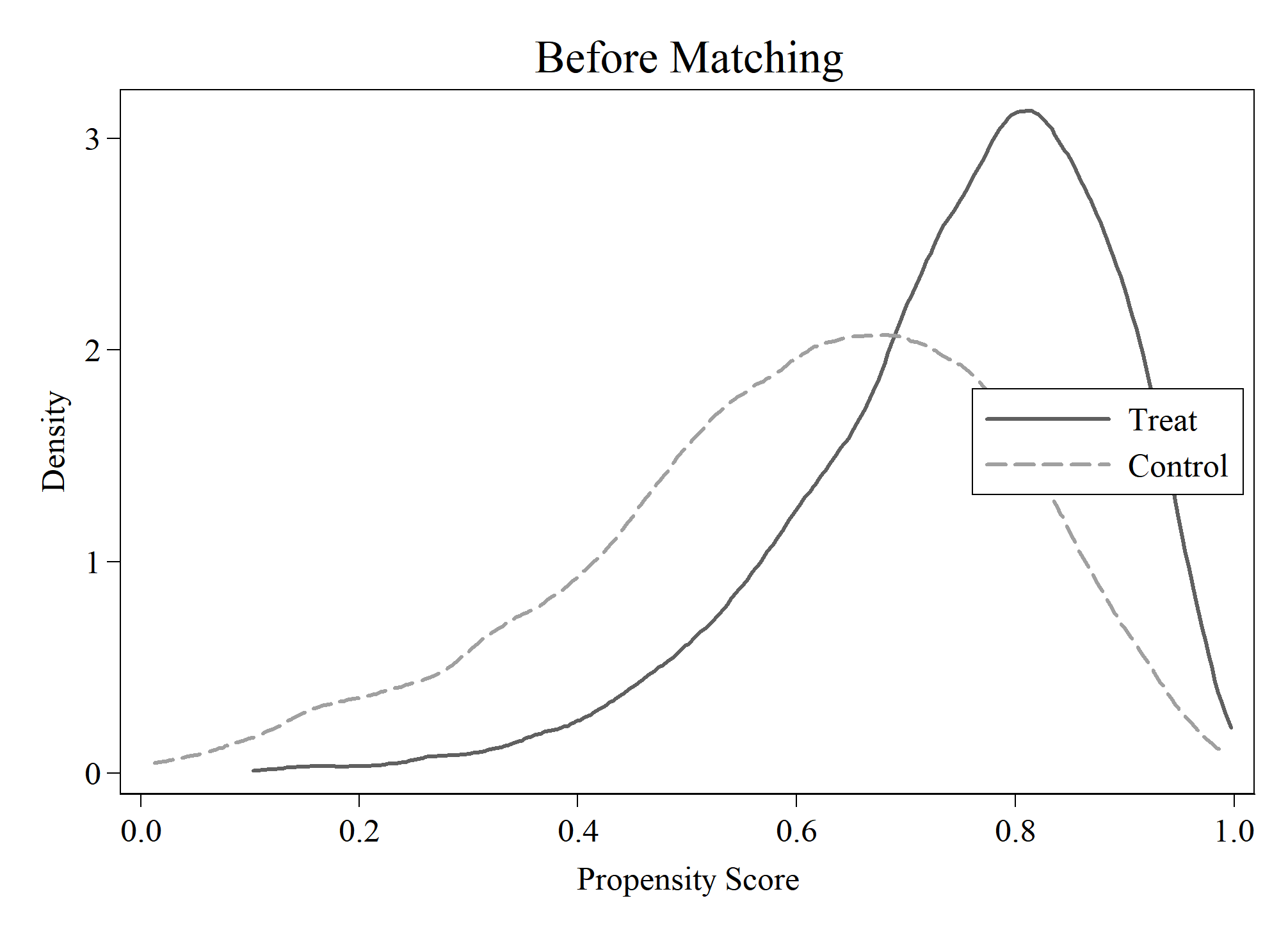


**Figure B1** Density before pairing


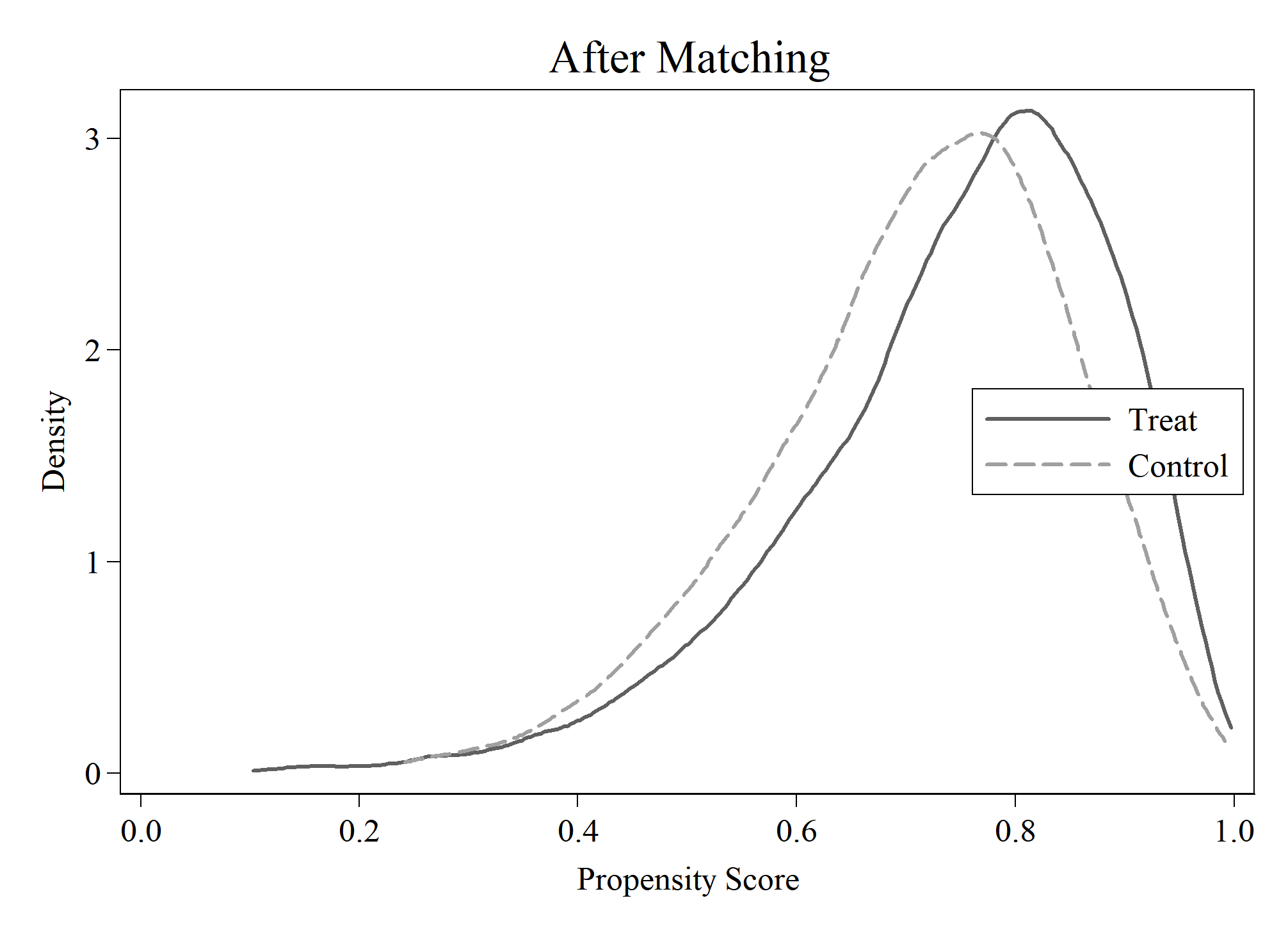


**Figure B2** Density after pairing
